# Biomechanics and Sports Performances

**DOI:** 10.3390/sports13030073

**Published:** 2025-03-04

**Authors:** Valerio Giustino, Antonino Patti

**Affiliations:** Sport and Exercise Sciences Research Unit, Department of Psychology, Educational Science and Human Movement, University of Palermo, Via Giovanni Pascoli, 6, 90144 Palermo, Italy; valerio.giustino@unipa.it

Biomechanics is the application of the principles of mechanics to humans; that is, the study of the motion of bodies and the causes that determine it [1]. Indeed, the study of the mechanics of movement in sports was born to allow the quantitative measurement of sporting gestures; that is, to measure the movement without considering the forces that made the movement possible (kinematics), or to measure the internal and/or external forces that determined the movement (kinetics) [2,3,4,5].

Sports biomechanics allows for athletes to learn the technical gestures and for coaches to detect and correct any executive defects [6]. To achieve these goals, the technology is increasingly coming to the aid of scientists and professionals, both for improving performance and for preventing injuries [7,8,9]. In particular, non-invasive instruments, such as the LiDAR technology capable of evaluating the morphology of the spine through a 3D scanning method, have proven to be valid in monitoring the athletes’ physical status in order to manage low back pain in athletes [10]. In a similar way, the infrared method of thermography could be effective to assess the thermal muscle response to spinal alterations [11]. Recent advancements have led to the development of portable and wearable devices that allow for the measurement of technical gestures away from laboratories, namely on the field [12]. For example, in running, the use of pressure insoles provides the advantage of collecting data on the vertical ground reaction forces in an actual in-game environment [13]. Similarly, inertial sensor technology is becoming more frequent for real-time performance analysis in combat sports [14].

In this Special Issue, titled “Biomechanics and Sports Performances”, 12 papers, including 11 research articles and 1 review, were published, with the aim of examining the applications of any training programs or the use of new technologies that can monitor the biomechanics of specific technical gestures in order to improve sports performance and reduce the risk of injuries.

The first paper of this Special Issue (Contribution 1) is a research article in which Bourantanis and colleagues aimed to explore the biomechanics in ancient Greek combat sports by analyzing pottery depictions. In detail, the authors, using computer vision technology, analyzed two postures (i.e., readiness and kicking postures) of the Pankration, or ancient Greek athletics. This technology is a multi-stage Convolutional Neural Network (CNN) that is able to process 2D images and generate keypoint data in order to construct static multi-segmental models, which allowed them to simulate the postures of the pottery depictions estimating some biomechanics parameters, such as joint forces and moments, weight distribution, and ground reaction forces. The main findings showed significant forces in the right leg, with the highest moment at the knee joint for the readiness posture, and as for the kick posture, the authors detected the highest moment at the knee of the supporting leg. Among others, this contribution underlines how it is possible to analyze static positions for providing quantitative estimates of joint forces and moments in combat sports.

The research by Dobos and colleagues (Contribution 2) was developed with the aim of studying the associations between the rate of force development (RFD) during a handgrip task performed in three different positions with the dominant arm, and the maximal post-impact ball speed (PIBS) of flat serves. Moreover, the authors aimed to study the correlations between the maximal voluntary contraction (MVC) of the dominant arm in the basic position and the PIBS. The MVC and RFD of the dominant arm during the tasks were measured in a sample of 23 elite junior tennis players. Specifically, the MVC of the dominant arm was assessed through an handgrip test in the basic position. Then, the RFD was measured during handgrip tests performed in three different positions (i.e., in the basic position, in the shoulder position, and in the flat serve position). Hence, the PIBS was estimated during a serve test. The results showed a significant positive correlation between RFD of the dominant arm in each of the three positions and the PIBS, and a significant positive correlation between the MVC of the dominant arm and the PIBS. The findings suggest that the rapid force generation of arm muscles may play a key role for a stable contact point, and that it has a relationship with the PIBS.

Vagner and colleagues (Contribution 3) investigated the relationships between body segment mass and combat techniques in a sample of sixteen male military cadets. The authors measured the body masses of different segments by using a dual-energy X-ray absorptiometry, the dynamic forces of different combat moves (such as punches, strikes, and kicks), and the performances of countermovement jumps. Although significant positive correlations were found between some body segment masses and specific combat gestures, these results suggest that knowledge of the body segment masses does not provide a significantly better estimate than knowledge of the body mass on the dynamic forces of combat moves.

Thorsen and colleagues (Contribution 4) aimed to assess the lower limb biomechanics in a sample of healthy adults in relation to increases in gait speed after a high-cadence cycling activities. To achieve this goal, the research group performed a kinematics and kinetics analysis of lower limbs during a walk at a self-selected pace before and immediately after 15 min of cycling activity at a cadence of 75 rpm. The main results showed that high-cadence cycling activity improves the gait speed by increasing the propulsive ground reaction forces, joint angular velocity during the swing phase, and positive power production during the stance phase. These findings suggest to promote cycling in order to increase gait speed.

The aim of the research article by Cartón-Llorente and colleagues (Contribution 5) was to analyze the impact of leg asymmetries on spatiotemporal running parameters carried out at different speeds (i.e., 12 and 14 km/h) for 3 min on a treadmill in under-14 runners. By using sensors attached to both shoes, the athletes showed high bilateral symmetry in the spatiotemporal parameters of running. Moreover, the bilateral asymmetries did not change between the two different speeds. This study added new knowledge on the assessment of leg asymmetries through wearable devices for the evaluation of its impact on sport performance and injuries in young athletes.

The study by Petri and colleagues (Contribution 6) aimed to establish anthropometric reference values for elite soccer players of both sexes. The anthropometric profiles of a large Italian sample (Series A soccer players, and general population as controls) were recorded according to the guidelines of the International Society for the Advancement of Kinanthropometry (ISAK). Reference anthropometric percentiles, such as body height, body mass, circumferences, skinfolds, breadths, and somatotype, were computed and stratified by player’s position on the field and sex. The results showed that soccer players have lower values in the sum of the skinfolds than sex-matched controls. As for the somatotype, the male soccer players were ectomorphic mesomorphs, and the females were balanced mesomorphs, while the male controls were endomorphic mesomorphs and the females were mesomorphic endomorphs. These findings provides anthropometric reference values for elite soccer players according to sex and position on the field, and could help practitioners with evaluations of body composition by avoiding the use of predictive equations or assumptions.

The aim of another research article published (Contribution 7) was to evaluate knee kinematics, by using a motion analysis system, of a fundamental movement in ballet, namely the Grand Plié, in twenty professional classical ballet dancers. Prior to the kinematics analysis, the authors assessed the functional abilities and the passive ranges of motion of the participants’ knees. During the execution of a complete circle of the Grand Plié, an excessive internal rotation during knee flexion was detected, increasing the risk of knee injuries. These results highlight the importance of kinematics in dance movements for preventing knee injuries.

Nakai and colleagues (Contribution 8) investigated the effects of external abdominal pressure supports, by using a device that can be pressurized and decompressed, on dynamic balance, measured by the modified star excursion balance test. The dynamic balance of each participant of the sample, composed of young adults, was measured in the following two conditions (48 h apart from each other): under external abdominal pressure support, and under non-abdominal pressure support. Firstly, the maximum abdominal pressure of each participant was measured. Then, a pressure corresponding to 30% of the mean maximum abdominal pressure was set for the star excursion balance test performed under the external abdominal pressure support condition. The maximum posterolateral, posteromedial, and composite values of the star excursion balance test were significantly higher in the abdominal pressure support condition than in the non-abdominal pressure support condition, suggesting the effectiveness of this technique for dynamic balance.

Santos and colleagues (Contribution 9) aimed to assess the thermal asymmetry and dynamic force parameters with 45% and 80% of the One-Repetition Maximum (1-RM) on a bench press in paralympic powerlifters. The thermography and dynamic force parameters were measured before and after a specific training session. No asymmetry was found in the skin temperature, and there were differences between the results before and afterwards. The authors found significant differences in asymmetry and in moments in mean propulsive velocity, maximum velocity, and power with 45% of the 1-RM. As for 80% of the 1-RM, the authors detected asymmetry, but no differences between moments.

The purpose of the research by Alimoradi and colleagues (Contribution 10) was to study the effects of soleus stretching on ankle flexibility, dynamic balance, and performance tests in female soccer players. The participants were randomized into a stretching group (i.e., gastrocnemius, quadriceps, and hamstrings), a stretching group with soleus stretching, and a control group. Before and after the stretching programs, lasting 4 weeks (with three sessions/week), the ankle range of motion, Y-balance test, drop jump test, dynamic knee valgus test, and Illinois Agility Running Test were carried out. Among the results, significant improvements in the ankle range of motion, Y-balance test, and drop jump test were found in both stretching groups compared to control group. The only stretching group with soleus stretching showed improvement in the Illinois Agility Running Test. These findings underline that adding soleus stretching into regular stretching programs can lead to higher performances.

In the last research article of this Special Issue (Contribution 11), the authors carried out a stabilometric test and electromyographic analysis of the “standing bird dog” exercise performed in static, dynamic, ipsilateral, and contralateral conditions using a motion capture system, wireless electromyography sensors, and a triaxial force platform. In dynamic conditions, some muscles (i.e., gluteus maximum, multifidus, lumbar erector spinae, and gluteus medius) achieved a mean activation level higher than the static conditions. Among the main results, in static conditions, balance was more challenging in the mediolateral direction, as compared to anteroposterior direction, while in dynamic conditions, balance was more challenging in the anteroposterior direction.

In this Special Issue a systematic review was also published (Contribution 12), in which authors compared the front kick and roundhouse kick according to different types of targets and experience levels. To conduct the systematic review, the research group followed the Preferred Reporting Items for Systematic Reviews and Meta-Analyses (PRISMA) guidelines. The literature search was performed using Web of Science, SportDiscus, and PubMed. The parameters considered were the maximum force, impact force, maximum velocity, angular velocity, and execution time. The authors included eighteen articles concerning the front kick and twenty-five articles concerning the roundhouse kick. The main results showed that the impact forces of the front kick were significantly higher than those of the roundhouse kick across novice, sub-elite, and elite athletes. The maximum foot velocities of the roundhouse kick were significantly higher compared to the front kick for the sub-elite and elite athletes. The elite athletes had a significantly higher knee extension angular velocity with the roundhouse kick than with the front kick. These results suggest that front kicks produce more force, while roundhouse kicks have a more rapid execution.
